# Measuring Blue Space Visibility and ‘Blue Recreation’ in the Everyday Lives of Children in a Capital City

**DOI:** 10.3390/ijerph14060563

**Published:** 2017-05-26

**Authors:** Amber L. Pearson, Ross Bottomley, Tim Chambers, Lukar Thornton, James Stanley, Moira Smith, Michelle Barr, Louise Signal

**Affiliations:** 1 Department of Geography, Environment & Spatial Sciences, Michigan State University, East Lansing, MI 48824, USA; bottoml4@msu.edu; 2 Department of Public Health, University of Otago, Wellington 6242, New Zealand; tim.chambers@postgrad.otago.ac.nz (T.C.); james.stanley@otago.ac.nz (J.S.); moira.smith@otago.ac.nz (M.S.); Michelle.barr@otago.ac.nz (M.B.); louise.signal@otago.ac.nz (L.S.); 3 Environmental Science and Policy Program, Michigan State University, East Lansing, MI 48824, USA; 4 Department of Social and Behavioral Sciences, Harvard T.H. Chan School of Public Health, Boston, MA 02115, USA; 5 School of Exercise and Nutritional Science, Deakin University, Melbourne 3125, Australia

**Keywords:** blue space, children’s environments, neighborhoods, mental health, cities

## Abstract

Blue spaces (water bodies) may promote positive mental and physical health through opportunities for relaxation, recreation, and social connections. However, we know little about the nature and extent of everyday exposure to blue spaces, particularly in settings outside the home or among children, nor whether exposure varies by individual or household characteristics. Wearable cameras offer a novel, reliable method for blue space exposure measurement. In this study, we used images from cameras worn over two days by 166 children in Wellington, New Zealand, and conducted content and blue space quantification analysis on each image (*n* = 749,389). Blue space was identified in 24,721 images (3.6%), with a total of 23 blue recreation events. Visual exposure and participation in blue recreation did not differ by ethnicity, weight status, household deprivation, or residential proximity to the coastline. Significant differences in both visual exposure to blue space and participation in blue recreation were observed, whereby children from the most deprived schools had significantly higher rates of blue space exposure than children from low deprivation schools. Schools may be important settings to promote equitable blue space exposures. Childhood exposures to blue space may not follow the expected income inequality trends observed among adults.

## 1. Introduction

Blue spaces have been defined as visible surface water (e.g., lakes, rivers, canals, coastal water) [1,2]. Blue space may promote positive mental health through opportunities for relaxation, reflection, and social connections or may promote physical health through activities such as swimming and recreation [3]. As such, there are three primary theoretical pathways through which blue space benefits health. One relates to the visual exposure to natural spaces as a calming backdrop and the subsequent mental health benefits. Another pathway relates to the “access” to and usage of blue spaces including engagement in physical activities in blue spaces, maintaining a healthy weight, lowering blood pressure, and obtaining other physical health benefits [4]. Lastly, accessibility to and usage of blue spaces may also serve to promote social connections, as blue spaces are venues for recreation (e.g., swimming, picnics, fishing), with concomitant benefits to both physical and mental health. Measurement of these blue space exposures have largely been limited to coastal proximity, self-reported usage of blue spaces, proportion of residential neighborhood occupied by blue space, distance from home to nearest blue space, and visibility of blue space from the home. Likewise, the majority of blue space health research has been conducted among adult populations.

Among adults, most studies have investigated the mental health benefits of living near the coast or living in a neighborhood with a high percentage of blue space coverage. A cross-sectional study found that those living nearer the coast in England had higher self-rated health, after adjustment for multiple measures of socioeconomic status [5]. In another cross-sectional study in the Netherlands, the proportion of blue space within 1 km of the home, compared to green space, was associated with lower anxiety/mood disorder and exhibited a larger effect on health after adjustment for education, income, and other covariates [6]. This finding was echoed in other cross-sectional research in New Zealand where increased exposure to blue spaces (but not green spaces) visible from the home was associated with lower levels of psychological distress, after adjustment for both area-level and individual-level socioeconomic status [7]. However, in the one longitudinal study published to date, no effects were detected between coastal proximity and life satisfaction and small effects for self-reported general and mental health [8]. These studies evaluate blue space exposures near the home and do not account for or estimate exposures that occur in other settings or in transit, or the contexts of blue space exposures.

Among the few studies to have explored these relationships in children, it has been found that visits to and living in close proximity to beaches are beneficial to children’s behaviors and to encouraging physical activity [9,10]. Among children in Barcelona, higher beach attendance was associated with lower behavioral problems but not Attention Deficit Hyperactivity Disorder symptoms [9]. As children and adolescents become increasingly autonomous of their parents and guardians, they begin to experience their neighborhoods through independent, unsupervised activities as well as through more “structured” activities managed by adults [11]. Blue spaces have been cited as being particularly important places to engage in play, with a long history of “blue exercise” and related child health benefits noted in the UK [12]. A coastal gradient in childhood obesity was detected for rural areas and small towns, but not cities in England [13]. One Norwegian study also found that childhood experiences in natural environments were associated with nature-based physical activity as an adult [14], meaning that blue space exposures in childhood may have lasting effects.

Most studies examining blue space exposure and mental health have been limited to adult populations, and related to exposures at home. However, the functional and structural changes occurring in the child and adolescent brain reflect a sensitive period in brain development. Evidence suggests that changes in the social environment that occur during adolescence may lead to heightened social sensitivity and influence a number of adolescent behaviors [15]. Thus, mental relaxation, opportunities for social connection, and the calming backdrop of blue spaces may be particularly salient to mental health and development in this population.

Although the evidence on potential benefits of blue space is still emerging, one of the limitations in this research has been a lack of quantification of *actual* visual exposure, with most studies using spatial proxies based on two-dimensional mapping of the amount of blue space near the home [6] or proximity to coastal environments [8], or self-reported instances of exposure or self-reported use of blue spaces [9]. Another limitation of previous studies is the lack of measurement of blue space exposures outside of the home, in transit or in relation to other places spend time, including work locations and schools. Furthermore, because there are few studies that have objectively measured blue space exposure, aside from residential distance to coastline, there is limited knowledge on the differing ways of quantifying such exposure and into the demographic factors associated with variations in exposure. Wearable cameras may offer a reliable method for quantifying actual exposure to blue space—both visually and in relation to blue space usage [16]. Understanding the nature and extent of blue space exposures may provide insight into possible inequality in exposure to blue space and to the potential physical and mental health benefits.

Using wearable cameras as a novel method to quantify blue space exposures, in a sample of children in Wellington, New Zealand, this study aimed to: (1) assess actual visual exposure to blue space in everyday activities, including those occurring outside the home; (2) objectively measure ‘blue recreation’ behaviors rather than relying on self-report; and (3) use these variables to explore the context of blue space exposures and to test whether exposures vary between children based on child characteristics including residential coastal proximity.

## 2. Materials and Methods

### 2.1. Study Participants

This study utilizes data from a larger study of the world in which children live and their interactions with it, called Kids’Cam. Kids’Cam is a cross-sectional observational study of the world in which children live. Sixteen out of 28 invited schools (57%) agreed to participate. A total of 192 out of 443 children (43%) consented to participate in the study. Of the 192 that provided consent, 168 (88%) were chosen to participate so as to avoid over-sampling from some schools. Participants were, thus, 168 randomly selected children, aged 11–13 years in the Wellington region of New Zealand. No information was collected for non-consenting children.

Each child wore a wearable camera for 4 days (two school days and the two weekend days). Data were collected over a one-year period. Ethical approval was obtained from the University of Otago Human Ethics Committee (Health) (13/220). During data collection, both students and staff were blinded to the blue space focus of the current study, as it is one of many studies generated from the data. For full details of the study methodology, see Signal et al. [16]. Wellington is the capital of New Zealand, an island nation, with large quantities of coastline.

### 2.2. Data Collection for Kids’Cam

The participants in the study wore wearable cameras (Autographer http://www.autographer.com) over a 4 day period (Thursday to Saturday). Participants were asked to wear the cameras for their entire waking day/evening. Cameras were configured to capture a 136° image every ~7 s, thus each image accounted for seven seconds of observation time. Children actually wore the cameras for a total of about 2500 h, leading to approximately 1.3 million total images over the study period (or approximately 31% of four 12-h days). Children were instructed to remove the cameras in situations where privacy could be expected, when swimming or playing sports, or if requested. Images were then compiled and processed.

Demographic characteristics for each child were also collected at enrollment, including sex, ethnicity (Pacific, Māori, and New Zealand European), household deprivation status, and school decile (a measure of school socioeconomic status). In addition, each geocoded house location was assigned a neighborhood deprivation score, based on the 2013 census data at the meshblock level (smallest unit of aggregation). This measure of neighborhood deprivation is called NZDep and is a widely used measure by both government agencies (e.g., [17]) and health researchers (e.g., [18]).

### 2.3. Image Data Processing for This Study

Image data for Thursdays and Saturdays (*n* = 749,389) from all 168 participants were extracted from the Kids’Cam dataset. One weekday and one weekend day were selected to maximize variation in settings where blue space exposure occurred, activities during exposure, time of exposure, and social contacts during exposure. Children with no images on these two days were excluded (*n* = 2), leaving a total of 166 children included in this study. Images taken between 7:00 a.m. and 7:00 p.m. on these two days (*n* = 691,606) were systematically examined by a researcher for the presence of blue space, which included natural blue space (e.g., rivers, streams and ocean) and human-built blue space (e.g., swimming pool). The study hours included in this study were selected to be relatively consistent “daylight hours” throughout a year.

If it was difficult to determine if an image contained blue space (due to blurriness or glare in the image (see Figures S1A, B)), then such images were reviewed by other researchers for consensus. In one instance, a child went into a public swimming pool, turned his camera off to swim, and then turned it back on when leaving. For this special circumstance, the number of images that would have occurred during this time were calculated as one blue space exposure, with 50% of the pixels in each image assumed to be blue space.

### 2.4. Quantification of Blue Space Exposure

Each image containing blue space was then assigned an exposure quantity, whereby blue space pixels were counted (more detail below). Image sequences separated by a break of more than 30 min between images containing blue space were considered separate exposures. Each exposure was then coded in an Excel file, noting the setting, activity (e.g., walking to school), and social contacts (e.g., alone, with peers, with adults, or mixed-age companions).

To assess visual exposure to blue space, images containing blue space were processed to quantify (per daylight hour observed) the following: (i) the number of images containing blue space; (ii) the minutes exposed to blue space (which is in effect the same numerical information as (i) but rescaled, given that each image is treated as being of constant duration); (iii) the number of exposures; and (iv) the number of blue space pixels in each image. To accomplish this, all images containing blue space (*n* = 5420) were counted. The duration of exposure in minutes was calculated by multiplying the number of images by 7 s (as the median inter-image interval from the cameras). The number of separate exposures were tallied. Then, each image was processed by hand (over 80 h) in order to count the number of blue space pixels in each image. Each applicable image was loaded into ArcGIS 10.3 software (ESRI, Redlands, CA, USA). A polygon was then digitized around all blue space and descriptive statistics were calculated to count blue pixels for each image.

To objectively measure ‘blue recreation’ behaviors or events, each activity code was further classified as occurring at home, at school, in transit, during recreation (general) or during blue recreation. Blue recreation was defined as personal engagement with water, prolonged camera focus on others engaging in water-based recreational activities, or camera focus on bodies of water for at least 30 continuous seconds during some form of recreation (including walking, picnicking, hiking, or biking along the coastline or a river) (see Supplementary Materials for further details on these designations). Last, a rate of blue recreation events per hour observed was calculated for each child.

### 2.5. Residential Distance to Coastline

Distance from each geocoded home location to coastline was calculated in meters, using ArcMap v10.3 (ESRI, Redlands, CA, USA). The median distance (2.4 km) was then set at the cut-off for high versus low. Sensitivity analyses were carried out using other thresholds, including the mean, 1 km, and 5 km, as conducted in other studies [8]. However, the differences in findings were minimal (not included).

### 2.6. Statistical Analyses

**Figure 1:**
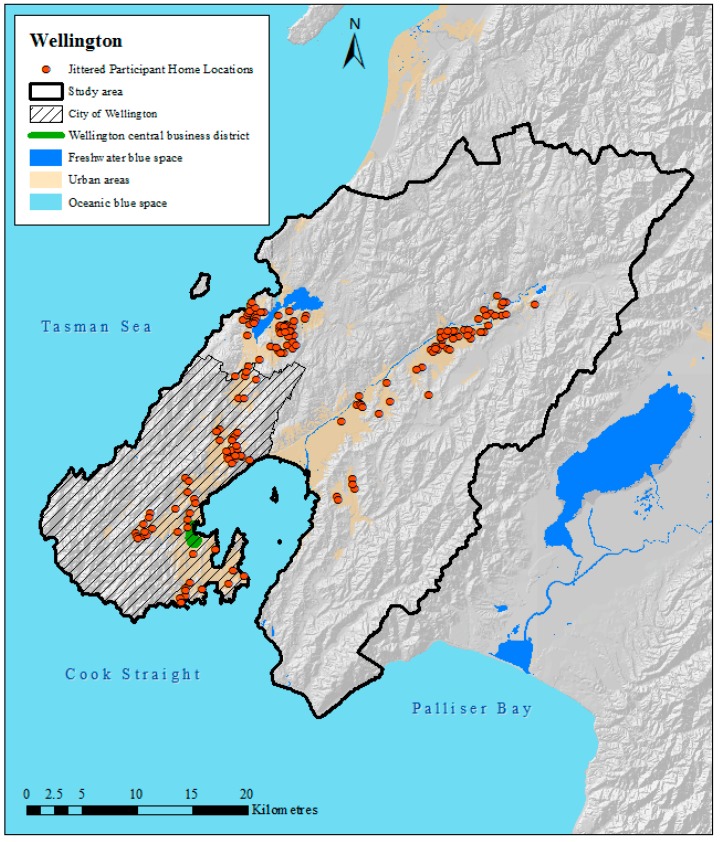
Map of study area, participants’ jittered home locations and blue spaces in Wellington, New Zealand.

Descriptive statistics of the study sample were first calculated for the study sample (*n* = 166), and home locations were mapped (Figure 1). Note that geocoded home locations of participants were jittered or geomasked to maintain confidentiality but to display the general spatial distribution of participants [19]. Jittered data were used for display only.

In order to compare various blue space exposure variables, descriptive statistics were calculated for each of the blue space exposure metrics (number of images, minutes exposed to blue space, number of exposures, blue space pixels, and blue space recreation events). Spearman’s rank correlations were used to assess similarities between these measures of blue space exposure. Next, we calculated mean blue space images per hour observed by day of the week, time of day, setting, and companion status to explore trends. Lastly, to explore the context and potential differences between sub-groups, rate ratios of both blue space images per hour observed and blue recreation events per hour observed (as two separate analyses) were calculated by ethnicity, sex, household, neighborhood and school deprivation status, and the child’s residential distance to the coastline. We selected these two measures of exposure to blue space, as one indicates visual exposure and one indicates behavior and physical activity related to blue space. To calculate means, 95% confidence intervals and rate ratios, zero-inflated negative binomial models were fitted, offset by the daylight hours observed, with zero-inflation by age. The complex sampling design for the Kids’Cam data collection [16] was accounted for in all inferential statistics (e.g., 95% confidence intervals, *p*-values) [20] using Stata’s svy prefix commands and associated weighting options using Stata v14 software (StataCorp, College Station, TX, USA).

## 3. Results

Table 1 includes the characteristics of the study sample. Our study sample had slightly more females than males (53% versus 47%). The sample included higher proportions of New Zealand European males and Māori females, which was a feature of the Kids’Cam sample. Over 30% of the participants were from homes with the lowest level of deprivation. The sample also included similar percentages of those from the lowest and the highest levels of neighborhood deprivation (23% and 25%, respectively). Participants were similarly distributed across school deprivation status, with slightly more students from the highest level of school deprivation. Neighborhood and household deprivation or overweight status were missing for a few participants. The subsequent analytical dataset was restricted to those for whom we had full data (*n* = 157).

**Figure 2:**
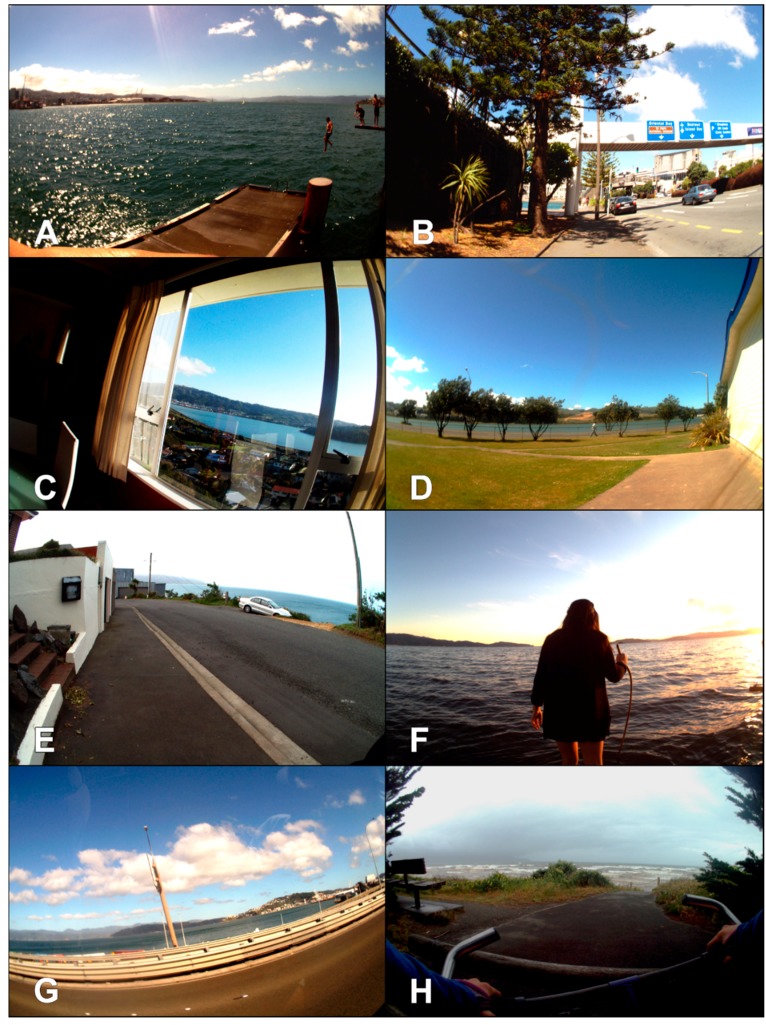
Example images for blue space exposures (**A**) blue recreation—swimming, (**B**) walking, (**C**) at home, (**D**) at school, (**E**) when alone, (**F**) with a peer companion, (**G**) in transit, and (**H**) blue recreation—biking.

Out of all the images processed (*n* = 691,606, equivalent to 1338.2 daylight hours), blue space was identified in 24,721 images (3.6%). Children were exposed to an average of 11.6 images/h containing blue space (95% CI: 5.3–18.0) for an average of 1.4 min/h (95% CI: 0.6–2.1) and 0.1 exposures/h (95% CI: 0.1–0.1). Children were exposed to 1.9 million blue space pixels (95% CI: 0–4.4 million). Children engaged in a total of 23 blue recreation events including hiking, biking along the coastline, swimming, horseback riding along a river, and taking a picnic at the water (see Supplementary Materials and Figure 2A–H for more details). On average, children engaged in 0.01/h blue recreation events (95% CI: 0.01–0.02).

On average, exposure to blue space was higher on Thursday, compared to Saturday, and in the morning, compared to the afternoon or evening (Table 2). Although not significantly different, on average, blue space images were most commonly from blue recreation activities (3.7 images per hour), followed by images from school, at home, during general recreation, and in transit. On average, children had higher exposure to blue space when with peers, compared to when alone or with adults or mixed aged companions, although these mean differences were not statistically significant, as assessed through overlapping confidence intervals. Figure 2 presents examples of images by setting and companion status. All of the measures of blue space exposure were strongly and significantly correlated with one another (rho = 0.47 to 0.98, *p* < 0.005), with the lowest correlation observed between the number of visual exposures and the number of blue space recreation events (Table 3). In addition, all blue space exposures (except blue recreation) were negatively and significantly correlated with residential distance to coastline. Although these correlations were weak to moderate (rho = −0.29 to −0.40, *p* < 0.005).

In exploring the differences in visual blue space exposures (counts of images containing blue space) based on child characteristics, similar rates were found across ethnic groups, by sex, weight status, and household deprivation status (Table 4). Surprisingly, significantly higher rates of exposure were detected among children living in the most deprived neighborhoods (IRR: 4.56, 95% CI: 1.64–12.66) and the middle deprivation neighborhoods (IRR: 2.66, 95% CI: 1.11–6.39) compared to the least deprived neighborhoods. Similarly, children attending high deprivation schools had significantly higher exposure to visible blue space (IRR: 5.47, 95% CI: 1.81–16.53), compared to children attending low deprivation schools. Rate ratios of visible blue space exposure were not significantly different for children residing near the coastline compared to children who were far from the coastline.

Similar trends were observed for rates of blue recreation events. However, the rate estimates were less precise, as the events were less common. Still, significant differences by school deprivation status were detected, whereby children from the most deprived schools (IRR: 5.90, 95% CI: 1.73–20.05) engaged in significantly more blue recreation events compared to those from the least deprived schools.

## 4. Discussion

This study aimed to quantify and understand trends in visual exposure to blue space and participation in blue recreation among children in the Wellington region of New Zealand. We found that, despite living in an island setting where exposure to blue space would be expected to be high, surprisingly, the children in this study were exposed to it only 3.6% of the observed time. We found no significant differences by ethnicity, overweight status, household deprivation, or residential proximity to coastline, with only weak to moderate correlations between exposure to blue space and residential proximity to coastline.

Within the sample of children from Wellington, surprising trends by neighborhood and school deprivation were detected, whereby children from more deprived neighborhoods or schools had higher rates of visual exposure to blue space (and higher rates of blue recreation by school deprivation only). Few studies have directly examined exposure to blue space but since higher land values are often observed in coastal areas [21], it was expected that both residential proximity to blue spaces and household deprivation would exhibit significant differences. This study, in fact, found the opposite to be true for schools and neighborhoods. In contrast, data from a study of Wellington adults identified significant correlations between higher exposure to visible blue space and both lower neighborhood deprivation and higher personal income (unpublished, using data from [7]). Thus, childhood may be an important time for equitable exposure to blue space benefits, as many exposures occurred during recreation (both blue and general) or at school. Indeed, schools that are considered more socioeconomically deprived in the Wellington region are also near the Porirua Harbor and Hutt River. For this reason, some caution must be used when generalizing this study’s results to other settings where there may be less egalitarian geography to the location of schools. Because blue space visibility at home or in transit is not likely to be amenable to change, increasing school time exposure to blue spaces either through recreation or simply getting within view of nearby blue spaces may be an important arena for equitable health promotion.

A key strength of this study was the systematic use of wearable cameras and subsequent digital images to quantify children’s visual exposure to blue space and blue recreation in their everyday lives. This methodology enabled a more robust, objective measurement of exposure than other methods that are more prone to recall bias (e.g., self-reported), and accessed the experiences of children, who are sometimes considered hard-to-reach as a study population [22]. Further, this method enabled the relatively unobtrusive observation of the contextual elements of children’s exposures to blue space. These contextual elements provide insight into the mechanisms of exposure that are harder or impossible to identify using other research methodologies. Still, it is unclear what level (length of time or quantity) of exposure to visual blue space, in adults or children, yields mental health benefits, which should be a priority for psychological research. Robust, objective measurement of mental health benefits of blue space is particularly important, as depression and mental health issues are now major contributors to the global disease burden [23].

Future studies could make use of eye-tracking technologies to quantify visual exposure to blue space. In addition, isolating the effects of different blue space exposure pathways on mental health would be a valuable contribution. For example, such studies could, by design, isolate the mental health benefits of visual (e.g., looking at the ocean through a closed window) versus auditory (e.g., listening to the ocean through an open window at night) exposure to blue space. However, anthropogenic noise may interfere with the possible mental health benefits and, thus, should be accounted for in such future studies [24].

In terms of linking blue space exposures to mental health, the most useful outcomes are likely indicators of stress (as opposed to severe outcomes like suicide), possibly using biomarkers, neuro-endocrine measurements or cognitive assessments rather than self-report. However, recent, parallel research on green space has shown associations between exposure and more severe mental health outcomes including depression and anxiety. This dose–response research found that neighborhood vegetative coverage of 20–30% yields lower stress, anxiety, and depression in dose–response analyses [25]. Similar robust studies on blue space health benefits are needed, though our study indicates that quantification of exposure to blue space needs to include more than residential neighborhood information.

A limitation of this study was that the images do not establish with certainty that a child is visually exposed to the elements detected in the images. For example, a child may be looking the other way when the blue space is recorded by the camera. Eye-tracking technologies would be one avenue to overcome this limitation. For this reason, this study conservatively claims that the visual exposures assessed in this study are “probable visual exposures” rather than “actual visual exposures”. Another limitation was the reliance on the user to both keep the wearable camera charged and to power it on/off each day during the study. Moreover, children’s compliance to the study protocol (not wearing cameras to protect third party privacy and protecting cameras by removing them during sporting activities) reduced the overall number of images captured, and may have led to unobserved blue recreation events. However, review of images leading up to camera removal meant this was unlikely. Therefore, compliance with the study protocol meant that the total possible observation time was reduced and the circumstances requiring removal of the camera typically occurred during daylight hours.

The number of cameras and researcher person hours led to a year-long data collection period. In order to account for seasonal differences in daylight, we chose study hours that consistently have daylight throughout the year (and accounting for daylight savings). While visual exposure to blue space is unlikely to vary greatly across seasons, cloudy or overcast conditions may reduce visibility of features in the distance. Engagement in blue recreation may be more likely to differ seasonally or during rain events. Because the Wellington region does not experience dramatic shifts in temperature across seasons (in 2016, the lowest monthly average was 10 °C; the highest, 24 °C [26]), this is expected to be minimal in this population of children. It would be useful for future studies to sample during a single season or across seasons, with data collection on temperature, precipitation, cloudiness, etc. In addition, this study’s sample size limited our ability to detect significant differences within or across groups, and the original study design was intended for assessing children’s exposures to food marketing. Future studies could increase sample size in order to incorporate more complex statistical analyses. Indeed, the current study has described patterns of everyday exposures to blue space among children, including by context, companion status, and locations, and thus has provided information that future studies can use. Another possible limitation could be an over-representation of images taken at school, which made up 46.1% of all study data, while school hours comprised only 25% of the study times (based on a single six-hour school day out of two 12 h days of data collection). This may relate to commitment of the school community to the research. Future studies could conduct similar studies to extend the generalizability of this study’s findings. Future studies among children may also benefit from considering proximity from school to coastline, in addition to residential distance to coastline. Importantly, the potential pathway between blue space exposure and health could be strengthened with experimental studies to quantify such potential health benefits among children.

## 5. Conclusions

Image analysis is a novel and reliable method for quantifying visual exposure to blue space and engagement in blue recreation. High rates of exposures during the weekday, in the morning, with peers, and while at school suggest that schools may be important settings to promote equitable blue space exposures. We also find that childhood exposures to blue space may not follow the expected income inequality trends observed among adults.

## Figures and Tables

**Table 1:** Sample characteristics in this study sample, *n* (%).

Characteristic	Category	*n* = 166
Ethnicity	New Zealand European	65 (39)
	Māori	59 (36)
	Pacific	42 (25)
Weight status	Overweight/obese	71 (43)
	Not overweight	94 (57)
	*Missing*	1 (0.1)
Household deprivation	1—Low	52 (31)
	2	33 (20)
	3	24 (15)
	4	26 (16)
	5—High	26 (16)
	*Missing*	5 (3)
Neighborhood deprivation	1—Low	39 (23)
	2	29 (17)
	3	26 (16)
	4	27 (16)
	5—High	42 (25)
	*Missing*	3 (2)
School deprivation	3—Low	51 (31)
	2	54 (33)
	1—High	61 (37)

**Table 2:** Mean count of blue space images, by day of week, time of day, setting, and companion status (estimates and 95% CI from zero-inflated regression models).

Context of Blue Space Exposure		Mean	95% CI
Day	Thursday	6.0	0.45–11.44
	Saturday	5.7	2.6–8.7
Time of day	Morning	5.7	0.8–10.6
	Afternoon	4.3	1.8–6.7
	Evening	1.7	0.3–3.0
Setting	School	2.7	0–7.3
	Home	1.9	0.1–3.6
	Recreation—general	1.6	0.5–2.7
	Transit	1.4	0–2.8
	Recreation—blue	3.7	1.1–6.3
Companion status	Alone	2.4	0.7–4.1
	Adults /+ mixed ages	3.4	1.2–5.6
	Peers only	5.4	0.8–9.9

**Table 3:** Spearman’s rank correlations between blue space exposure measures and residential distance to coastline.

Blue Space Exposure Variables	Blue Space Pixels	Minutes Exposed to Blue Space	Count of Visual Exposures	Images Containing Blue Space	Residential Distance to Coastline
Blue space pixels	1				**−0.29**
Minutes exposed to blue space	**0.98**	1			**−0.37**
Count of visual exposures	**0.95**	**0.98**	1		**−0.40**
Images containing blue space	**0.98**	^†^	**0.98**	1	**−0.37**
Count of blue space recreation events	**0.60**	**0.54**	**0.47**	**0.54**	−0.01

Bolded items significant at the *p* < 0.005 level. ^†^ These measures are identical, but on different scales.

**Table 4:** Rate ratios for blue space images and blue recreation events per hour observed (from zero-inflated negative binomial regression models), by child characteristics. Separate univariate models fitted for each characteristic and blue space exposure measure.

Child Characteristics *Total n = 157*		Blue Space Images Rate Ratio (95% CI)	Blue Recreation Events Rate Ratio (95% CI)
Ethnicity	New Zealand European	1 (Reference)	1 (Reference)
	Māori	1.11 (0.38, 3.26)	1.82 (0.59, 5.59)
	Pacific	1.37 (0.29, 6.52)	2.99 (0.85, 10.52)
Sex	Male	1 (Reference)	1 (Reference)
	Female	1.12 (0.56, 2.27)	2.01 (0.95, 4.21)
BMI	Not overweight	1 (Reference)	1 (Reference)
	Overweight/obese	0.67 (0.23, 1.90)	0.89 (0.22, 3.50)
Household deprivation status	1—Low	1 (Reference)	1 (Reference)
	2	1.11 (0.44, 2.83)	1.67 (0.48, 5.81)
	3	1.22 (0.75, 1.99)	0.45 (0.05, 4.23)
	4	1.46 (0.50, 4.28)	1.05 (0.15, 7.18)
	5—High	2.19 (0.34, 14.07)	2.58 (0.03, 193.96)
Neighborhood deprivation status	1—Low	1 (Reference)	1 (Reference)
	2	0.82 (0.27, 2.44)	0.19 (0.01, 2.51)
	3	2.66 (1.11, 6.39)	1.10 (0.18, 6.69)
	4	1.88 (0.74, 4.78)	0.93 (0.20, 4.37)
	5—High	4.56 (1.64, 12.66)	3.30 (0.97, 11.26)
School deprivation status	3—Low	1 (Reference)	1 (Reference)
	2	1.54 (0.59, 3.99)	1.95 (0.72, 5.27)
	1—High	5.47 (1.81, 16.53)	5.90 (1.73, 20.05)
Distance to coast	Near	1 (Reference)	1 (Reference)
	Far	0.37 (0.09, 1.55)	0.96 (0.45, 2.07)

## Data Availability

Confidentialized data may be requested from the corresponding author, which includes no identifiable information about participants.

## Supplementary Materials

The following are available online at www.mdpi.com/1661-7827/14/6/563/s1, Table S1: Results of classification of blue recreation, Figure S1: Examples of images deemed unfit for processing (A) due to glare or (B) due to blurriness.
