# Supplementary material for: Measuring Blue Space Visibility and ‘Blue Recreation’ in the Everyday Lives of Children in a Capital City
Source: Int J Environ Res Public Health. 2017 May 26;14(6):563. doi: 10.3390/ijerph14060563 (PMC5486249; doi:10.3390/ijerph14060563)
Supplement: Supplementary file 1 [file ijerph-14-00563-s001.pdf]

Supplementary Materials

In this document, we outline the definition and method for classifying activities in images as either ‘blue recreation’ or not. In addition, we provide examples of images that were considered impossible to process for blue space.

I. Blue Recreation Classification

1. Definition: Blue recreation was defined as personal engagement with water, prolonged camera focus on others engaging in water-based activities, or camera focus on bodies of water for at least 30 continuous seconds during some form of recreation (including walking, picnicking, hiking, or biking along the coastline or a river).
2. Method of image sorting for activities: Blue space exposures were grouped into eight categories based on the activities that the participants were engaged in during the images. These categories include the following:

- i. At Home: exposures that took place inside the participant’s home or on their property. Exposures at home were not considered blue recreation—unless camera focus was maintained on water for at least 30 s, in which case it was coded as sightseeing (in one instance only, where the child sat to gaze at the water for an extended period).
- ii. At School: exposures that took place on school grounds (e.g. in class, during scheduled play, waiting to be picked up, etc.). Because blue space features were never located on school grounds and participants’ camera focus was never held on water, these exposures were not considered blue recreation.
- iii. In Car: exposures that took place within the family/friend’s vehicle. Because participants had no control over where they went or what they saw while in vehicle, and because the child was not engaged in recreation per se, no such exposures were considered blue recreation.
- iv. In Public Transit: exposures that took place on school buses, city buses, or trains. Treated the same as “In Car” just a different setting.
- v. Walking: similar to “In Car” or “In Public Transit,” but involved pedestrian transit. However, if walking along the beach for recreation, or if water was the focus of the walking activity, the exposure coded as “Sightseeing”.
- vi. Outdoor Recreation: instances of outdoor recreation that did not focus on water. For example, biking, hiking, or visiting a public park. These activities could be considered ‘land recreation’. These outdoor recreational activities were not conducted on or near the water and never involved reaching a water view point as a main part of the activity.
- vii. Sightseeing: exposures that involved camera focus on bodies of water for at least five consecutive images (roughly 30 s) but without direct contact with water. These exposures took place in outdoor settings and involved recreation events but were not directly on the water (e.g. sitting on beach, looking off of a hill to a water body).
- viii. Water-Specific Activities: exposures that involved the direct contact with water for recreation, or watching others engaging in water for recreation (e.g. at a waterpark, watching a swim meet, etc.). These events were always categorized as blue space recreation.

3. Method of Classification:

The main method used in classification was identifying the activities that participants were engaged in, as well as the duration of those activities. If exposures to blue space occurred within certain parameters that made it clear that the exposure was incidental (i.e. very low pixel count, blue space relegated to background of image, camera attention clearly focused on other stimuli), then exposures were treated as non-blue space recreation and categorized by activity. If the participant was actively engaged with the water for recreational purposes, or maintained camera focus on someone else engaging with water for recreation (i.e. swimming, wading, at locations focused on

water such as waterparks or pools) for at least 30 s (or 5 consecutive images), then the exposure was treated as blue space recreation and coded as "Water-Specific Activities." If the participant did not engage with the water, nor watch anyone else engage with the water, but did maintain camera focus with the body of water for at least 30 s (or five consecutive images), then the exposure was treated as blue space recreation and coded as "Sightseeing."

4. Results of Classification

Table S1. Results of classification of blue recreation

Activity	Instances	Considered blue recreation?	Reasoning	Examples
At Home	16	0	Blue space exposures at home were always incidental unless they specifically watched the water, then coded as sightseeing	Watching TV, socializing with family, eating meals
At School	24	0	Blue space exposures at school were never water oriented	In classroom, at recess, walking between buildings
In Car	9	0	In transit	Family vehicle
In Public Transit	3	0	In transit	City bus, school bus, train
Walking	62	0	In transit	Walking as a means of transportation (not along coastline)
Outdoor recreation	6	0	Outdoor recreation that was not water-centric	Hiking or biking (not along coastline)
Sightseeing	12	1	When focus on water bodies was maintained for at least 5 images (roughly 30 seconds), while engaged in outdoor activity	Hiking or biking along coastline or in order to get a view of the water, picnic on the water, walk along boardwalk, sitting on beach
Water-Specific Activities	11	1	Actively engaged with water or watching others engage with water	Horseback riding along river, water park, swimming

II. Example Images Deemed Unfit for Processing

Below are two images deemed unfit for processing to determine blue space. In one image, glare obscures the image (Figure S1A). In the other image, blurriness obscures the image (Figure S1B). These types of images constitute about 4% of all images processed in this study.

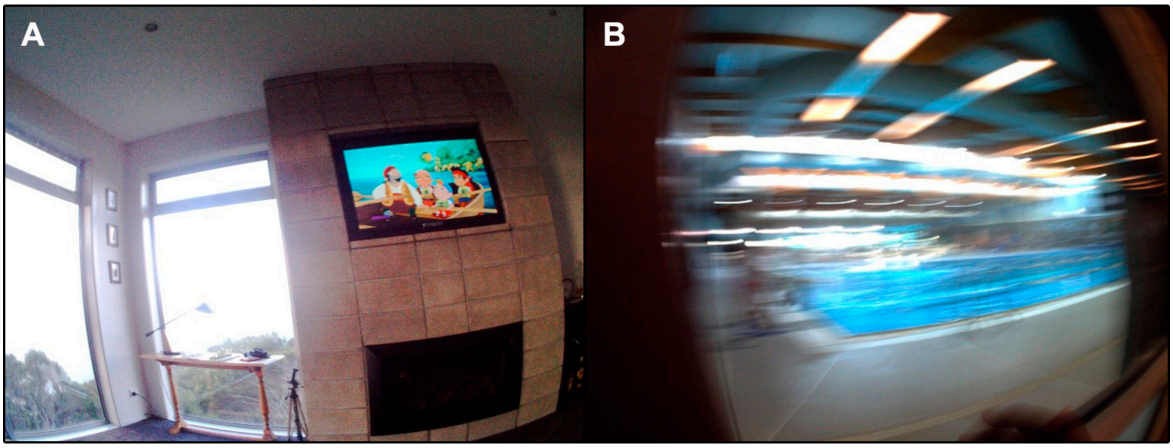

Figure S1. Examples of images deemed unfit for processing (**A**) due to glare or (**B**) due to blurriness.
